# Leveraging bioinformatics approaches for drug repositioning in space radiation protection

**DOI:** 10.1371/journal.pone.0357889

**Published:** 2026-09-15

**Authors:** Thomas E. Diaz, Joseph A. Kerrigan Jr, Dawn E. Bowles, Yared H. Kidane

**Affiliations:** 1 Eshelman School of Pharmacy, The University of North Carolina at Chapel Hill, Chapel Hill, North Carolina, United States of America; 2 Department of Pharmacy, The Johns Hopkins Hospital, Baltimore, Maryland, United States of America; 3 Research and Development, Twist Bioscience, Wilsonville, Oregon, United States of America; 4 Department of Surgery, Duke University Medical Center, Durham, North Carolina, United States of America; 5 Center for Pediatric Bone Biology and Translational Research, Scottish Rite for Children, Dallas, Texas, United States of America; Shiraz University, IRAN, ISLAMIC REPUBLIC OF

## Abstract

The health effects of space radiation, primarily Galactic Cosmic Rays (GCRs), on humans remain largely unknown, with potential cardiovascular consequences posing a significant threat to astronauts on long-duration spaceflight missions. Currently, there are no established pharmacological countermeasures for GCR exposure. Drug repositioning offers a promising strategy to accelerate pharmaceutical research in space medicine. This study leverages existing bioinformatics techniques to identify and prioritize potential drug candidates associated with proteomic perturbations following simulated GCR exposure using previously published murine cardiac proteomic data. A protein-protein interaction (PPI) network was constructed using the top differentially expressed proteins (DEPs) from murine heart tissue following exposure to 5-ion GCRs as seed nodes, focusing on experimentally supported interactions. Network topology, Markov clustering, and functional enrichment analyses were used to characterize biologically relevant proteins and pathways. Drug-protein interactions were predicted using Drugst.One and mapped to PPI clusters of interest to identify candidate drugs. Selected drug-macromolecule interactions were further explored using CB-Dock2 molecular docking and short-duration molecular dynamics simulations as hypothesis-generating structural assessments. Analysis of a key PPI network cluster consisting of several ATP synthase proteins identified 23 unique drug candidates. These analyses demonstrate a systematic approach for leveraging bioinformatics techniques to identify candidate molecular targets and generate pharmacological hypotheses in the context of space radiation countermeasures. Ultimately, this strategy introduces a hypothesis-generating framework for the prioritization of potential drug candidates for future computational characterization and experimental investigation against spaceflight stressors.

## Introduction

Space is an extreme environment that may pose unforeseen risks to the health and performance of those who undergo spaceflight. Of the few human studies conducted in space by the National Aeronautics and Space Administration (NASA) and its international counterparts, evidence of biological and physiological changes has been recorded [1]. The etiology of these changes are largely unknown, however, there are a plethora of unique stressors that are proposed to be responsible. Some of these stressors include space radiation, isolation, distance from Earth, gravitational alterations, and harsh environments, among others [2]. The effects of space radiation on the human body are difficult to elucidate because of the many aforementioned confounding factors in the space environment, but NASA has identified it as a critical area to explore due to potentially negative consequences to astronaut health. Although terrestrial radiation exposure in itself is a known risk factor for cardiovascular disease, exposure to space radiation may induce alterations that have yet to be understood. Researchers on Earth have utilized simulated space radiation in analog studies to explore the implications of this unique stressor [3]. These studies allude to space radiation induced carcinogenesis and ocular, bone, central nervous system, immune system, and cardiovascular alterations [2].

Efforts in recent investigations to mimic the space radiation environment through exposure to sequential Galactic Cosmic Rays (GCR) components have found evidence of cardiovascular damage [4,5]. For example, Bishawi et al. exposed murine models to several GCR components simultaneously as a single dose rather than individual mono-energetic ions at the NASA Space Radiation Lab (NSRL) [4]. Bishawi et al. reports deterioration and maladaptive changes in cardiac structure and function approximately one year after a dose of 150 cGy of GCR_5-ion_ exposure [4]. A secondary analysis of this study was recently published in Kidane et al., which found perturbed, inflammation-related, proteomic pathways in the heart and plasma [5]. Kidane et al. alludes to acute and chronic health issues after a single exposure to GCR_5-ion_, and supports the findings from Bishawi et al. [5].

There are few published studies exploring countermeasures for space radiation exposure, but a gap in knowledge still exists [6–11]. Drug repurposing, or repositioning, is the identification of new indications or targets that differ from a drug’s putative pharmacological effect [12,13]. This may be especially useful in light of “Eroom’s law,” a principle that highlights the increasing complexity and costs associated with drug discovery for rare diseases [13]. There are many tools that exist to aid in the drug repurposing process ranging from web-based servers to complex network-based software [12]. Each tool utilizes predictions of drug-target interactions, drug-disease interactions, or drug-induced gene expression profiles to present potential new uses for previously existing drugs [12]. Network-based analysis, or pathway mapping, is a widely used strategy for computational drug repositioning in which drug-target, drug-disease, or protein interaction networks assist in identifying therapeutic targets of action [13,14]. Within pathway mapping, betweenness centrality and node degree are useful network topological parameters for identifying “hotspots” within complex protein pathways [15]. The betweenness centrality of a node or edge within a network is a measure of the number of shortest paths between the node and any other given node, while the degree of a node is a measure of how much the node is integrated in the network (i.e., the number of interactions with other nodes) [15]. These parameters may be used to identify key proteins that have a strong influence on neighboring proteins within a protein-protein interaction (PPI) network.

In addition, molecular docking has been described as a useful method for exploring potential ligand interactions with targets of interest [16]. A structure-based approach, which may include free energy binding estimates and molecular dynamics, can provide hypothesis-generating information regarding predicted protein-ligand conformations and the behavior of selected complexes [16]. In the context of drug repositioning, docking is a commonly used strategy for screening potential therapeutic drug targets with drugs of interest [16]. For example, Kinnings et al. used molecular docking to identify entacapone, a US Food and Drug Administration (FDA) approved drug for Parkinson’s disease, as a drug candidate against resistant strains of tuberculosis [17]. This is one of many examples of the utility of molecular docking for exploring potential drug-target relationships.

Given the lack of human data from spaceflight, coupled with space radiation as a hypothesized risk factor for cardiovascular degeneration, the need for space radiation countermeasures is evident. Therefore, the exploitation of the aforementioned computational strategies alongside network topological values may prove to be useful in the realm of space medicine due to the complexity and safety risks associated with inflight pharmaceutical research studies. A recent study analyzed RNA sequencing data from human induced pluripotent stem cell-derived cardiomyocytes to identify cardiovascular-related pathways, and subsequently applied computational drug repurposing methods to suggest a list of drug candidates [18]. This demonstrates the utility of bioinformatics approaches to reposition drugs for spaceflight-induced perturbations.

This study is a secondary analysis utilizing proteomic data from previously published studies [4,5]. We aim to leverage existing bioinformatics techniques to identify and prioritize pharmaceutical candidates predicted to interact with proteins perturbed by GCR_5-ion_ exposure, providing a hypothesis-generating computational framework for future space medicine studies.

## Methods

Heart tissue of 6-month-old, male C57BL/6 mice were collected approximately 8 months after exposure to GCR_5-ion_ and compared to sham-irradiated mice. The methods of this collection process are described in detail in Bishawi et al, and this analysis builds on our groups’ previously published proteomics dataset [4,5]. Differentially expressed proteins (DEPs) from mouse heart tissue were identified and organized with their corresponding gene names [5]. Significance was defined as an adjusted p-value ≤ 0.05 and an absolute Log2 fold-change ≥ 0.263 (≥1.2-fold). Using these criteria, 87 of the 432 quantified proteins were significant. The majority (≈72%) exhibited modest changes below 1.3-fold. We therefore focused on the remaining ~28%, corresponding to the top 20 proteins, which showed absolute Log2 fold-changes from 0.397 to 1.462 (≈1.32–2.75-fold), representing the strongest signals. **S1 Fig** illustrates the distribution of Log2 fold-changes for all significant proteins, highlighting the top 20 at the extremes. These top 20 DEPs were each screened for a human ortholog using the National Center for Biotechnology Information (NCBI) gene database, HUGO Gene Nomenclature Committee (HGNC) database, and UniProt.

### Protein-protein interaction network

A network-based approach was employed to determine the relationships between the perturbed DEPs with their neighboring proteins and associated drug candidates. For this network-based analysis, the top 20 proteins served as seed nodes in a protein–protein interaction network. Signal propagation identified additional connected proteins, expanding the candidate list while remaining anchored to the most pronounced changes from our prior study. A curated PPI network was integrated using the Search Tool for the Retrieval of Interacting Genes/Proteins (STRING) version 12.0 [19]. The human orthologs of the top 20 DEPs following GCR_5-ion_ exposure were queried in STRING under the “multiple proteins” option, with *Homo sapiens* selected as the organism. In the STRING settings, “active interactions sources” were restricted to “experiments,” using the highest confidence score (0.900). The maximum number of interactors was set to 100 for both the first and second shell. Nodes with no identified interactions (“singletons”) were removed. The network was extracted as a tab-separated values (TSV) format file and uploaded to Cytoscape version 3.10.2 [20]. A network analysis was performed using the “analyze network” tool within Cytoscape to identify topological parameters within the node table. Betweenness centrality and node degree were chosen a priori as the primary topological parameters based on their ability to capture “hot spot” nodes that are likely to influence neighboring proteins. Other network metrics exist, though many emphasize peripheral or interlinked nodes, which may not be relevant in the context of drug-target prioritization in this population. The Cytoscape plugin clusterMaker2 version 2.3.4 was utilized to cluster the PPI using the Markov Clustering (MCL) Algorithm, with a granularity parameter of two, an array source of “experimentally determined interactions,” and an edge cutoff of 0.9 [21].

### Gene ontology (GO), pathway, and disease over representation analysis

The PPI proteins extracted from the TSV file format were uploaded to the Database for Annotation, Visualization and Integrated Discovery (DAVID) platform to access GO, Kyoto Encyclopedia of Genes and Genomes (KEGG) pathways, and DisGeNET disease associations [22,23]. GO terms, KEGG pathways, and DisGeNET disease associations were sorted in descending order using a threshold false discovery rate (FDR) of 0.01. The GO analysis included biological processes, cellular components, and molecular function. The top 10 GO terms, KEGG pathways, and DisGeNET disease associations were uploaded to an online bioinformatics platform for data analysis and visualization and plotted separately [24]. This step was performed to assess the biological context of the PPI network and interrogate whether its functional enrichment was consistent with cardiac pathways previously identified using the same proteomic dataset in Kidane et al [5].

### Drug-protein interaction network

The TSV file format containing the nodes and edges of the PPI was used to curate a drug-protein interaction network using the Drugst.One standalone tool, a drug repurposing web tool with interactive network visualization [25]. The PPI proteins were queried in Drugst.One using five embedded drug-target databases: (i) DrugBank; (ii) DrugCentral; (iii) NeDRex; (iv) DGIdb; (v) ChEMBL. For each query, the “autofill interactions” option was disabled to prevent the creation of new protein-protein interactions. The five resulting drug-protein interaction networks were extracted from Drugst.One as a GraphML file format and individually uploaded to Cytoscape as separate networks. Using the Cytoscape merge tool, the five drug-protein interaction networks were combined via an advanced network union merge. All duplicate edges and self-loops were removed.

### Analysis of drug candidates

The list of drugs from the drug-protein interaction network was cross-referenced with the protein clusters in the PPI generated using the MCL Algorithm. Specifically, drugs predicted to target proteins within a protein cluster of interest were identified for further analysis. Each drug was individually queried in PubChem (https://pubchem.ncbi.nlm.nih.gov/) to identify its canonical SMILES formula and three-dimensional conformer structure. The three-dimensional conformer structures were extracted as a Structure Data File (SDF) file format. The SMILES formula of each drug was input into the MolSoft drug-likeness and molecular property prediction database (https://molsoft.com/mprop/mprop.cgi) to obtain information about their molecular properties. This step was performed to determine whether any drug candidates violate Lipinski’s rule of five.

### Molecular docking

To explore potential interactions between the predicted drug candidates and target macromolecules, CB-Dock2 was utilized, a protein-ligand docking tool that employs curvature-based cavity detection and AutoDock Vina-based docking procedures [26,27]. This provided a hypothesis-generating estimate of possible drug-protein binding formations. Each protein from the protein cluster of interest was queried in the Research Collaboratory for Structural Bioinformatics (RCSB) Protein Data Bank (PDB) to obtain its three-dimensional structure (http://www.rcsb.org/). Experimentally determined structures available in PDB were used to maintain the analysis within experimentally resolved structural data to avoid introducing computationally modeled protein conformations. Since these cluster proteins exist as subunit entities within a macromolecular complex, the corresponding macromolecules were extracted in PDB file format. A drug was docked with a macromolecule only if network analysis predicted multiple protein interactions within the macromolecule or if it contained a protein among the top five betweenness centrality. Macromolecule and drug structures were not manually altered or pre-processed, and CB-Dock2 automatically prepared the structures by optimizing their geometry and hydrogen bonds. Default parameters were used for all docking simulations and were conducted via the structure-based auto blind docking tool for five binding cavities. The predicted quality of drug-protein docking was assessed based on its Vina score and cavity volume, with molecular dynamics simulations subsequently performed as an exploratory assessment of selected predicted complexes.

### Molecular dynamics simulations

Five drug-protein complexes were extracted for molecular dynamics simulation based on multi-criteria incorporating Vina score, cavity volume, the number of predicted drug-protein interactions within the macromolecule, and existing pharmacological evidence. Ligand topologies were generated using ACPYPE (v2023.10.27) with the GAFF2 force field and AM1-BCC partial charges. Protein topologies were generated using the GROMACS (v2025.4) pdb2gmx module with the Amber99SB-ILDN force field. Each complex was solvated in a cubic TIP3P water box with a minimum 0.8–1.0 nm padding from the protein surface, and the system charge was neutralized with Cl- ions. Energy minimization was performed using steepest descent with a maximum force tolerance of 1000 kJ/mol/nm. Systems were then equilibrated sequentially under NVT (100 ps, V-rescale thermostat, 300 K) and NPT (100 ps, Berendsen barostat, 1 bar) conditions with hydrogen bond constraints applied via the LINCS algorithm. Production MD simulations were performed for 5–10 ns using a 2 fs time step, C-rescale barostat, PME electrostatics with a 1.0 nm cutoff, and GPU acceleration locally on an NVIDIA RTX 4050 under WSL2. Binding free energies were calculated using the MM-GBSA method implemented in gmx_MMPBSA (v1.6.3) with the generalized Born model (igb = 5) and a salt concentration of 0.15 M, evaluated over 100–200 frames extracted from the production trajectory.

## Results

### Study design

**Fig 1:**
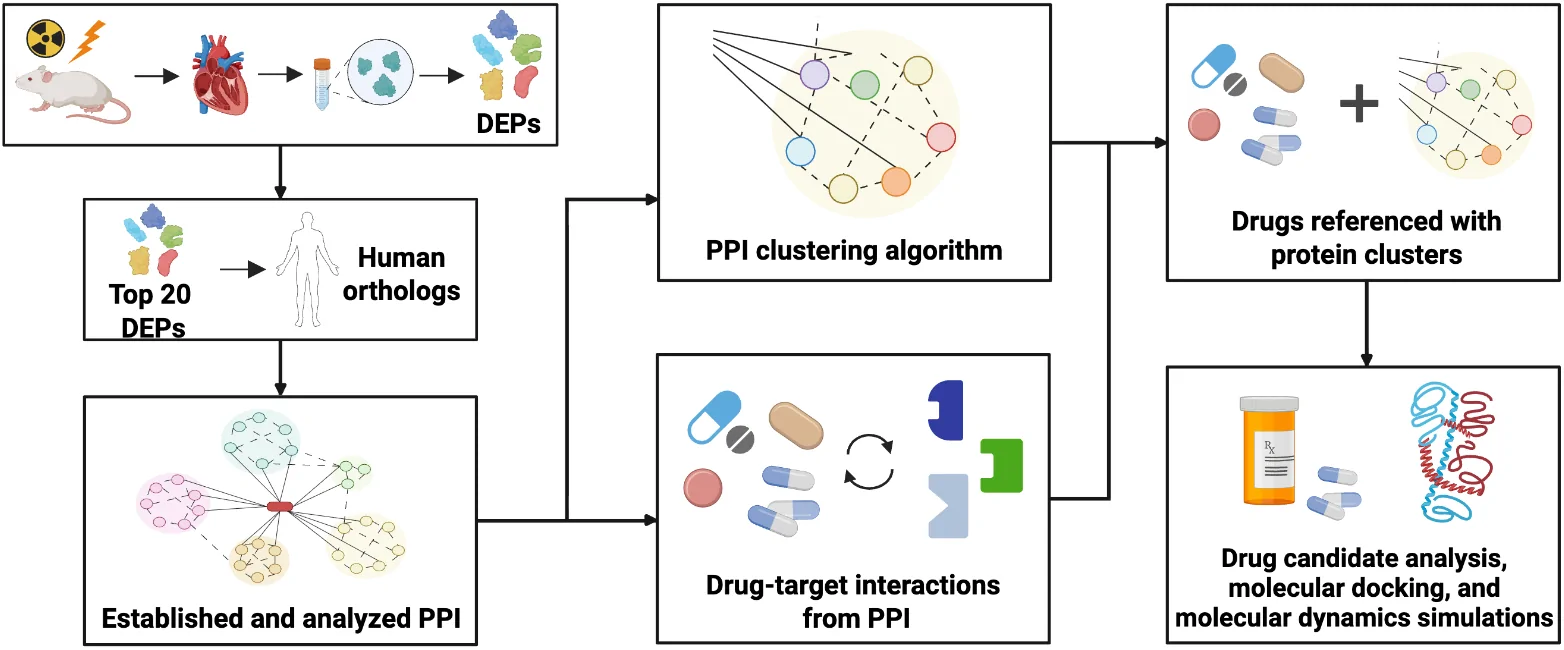
Flowchart of methodology. Flowchart of the computational drug repositioning workflow. Murine models were exposed to GCRs at the NASA Space Radiation Laboratory and heart tissue were collected for analysis. DEPs from the mouse heart tissue were identified, and a protein-protein interaction network was established based on experimentally determined interactions. Network clustering and topological parameters were used to identify influential clusters and proteins. Drug-protein interactions were identified using a drug repurposing web tool, then mapped to the clustered interaction network. Potential drug candidates were extracted and docked with predicted macromolecules associated with the interaction network, followed by molecular dynamics simulations. Created in BioRender. Bousquet, A. (2026) https://BioRender.com/gqlldbm.

The procedure utilized in this study is visualized in Fig 1 and is as follows: (i) established a protein-protein interaction (PPI) network using the DEPs; (ii) applied a clustering algorithm to the PPI; (iii) mapped the PPI and its clusters with drug-protein interactions from a drug repurposing web tool; (iv) extracted select drug candidates for analysis, molecular docking, and molecular dynamics simulations with select predicted macromolecules.

### Protein-protein interaction network

**Fig 2:**
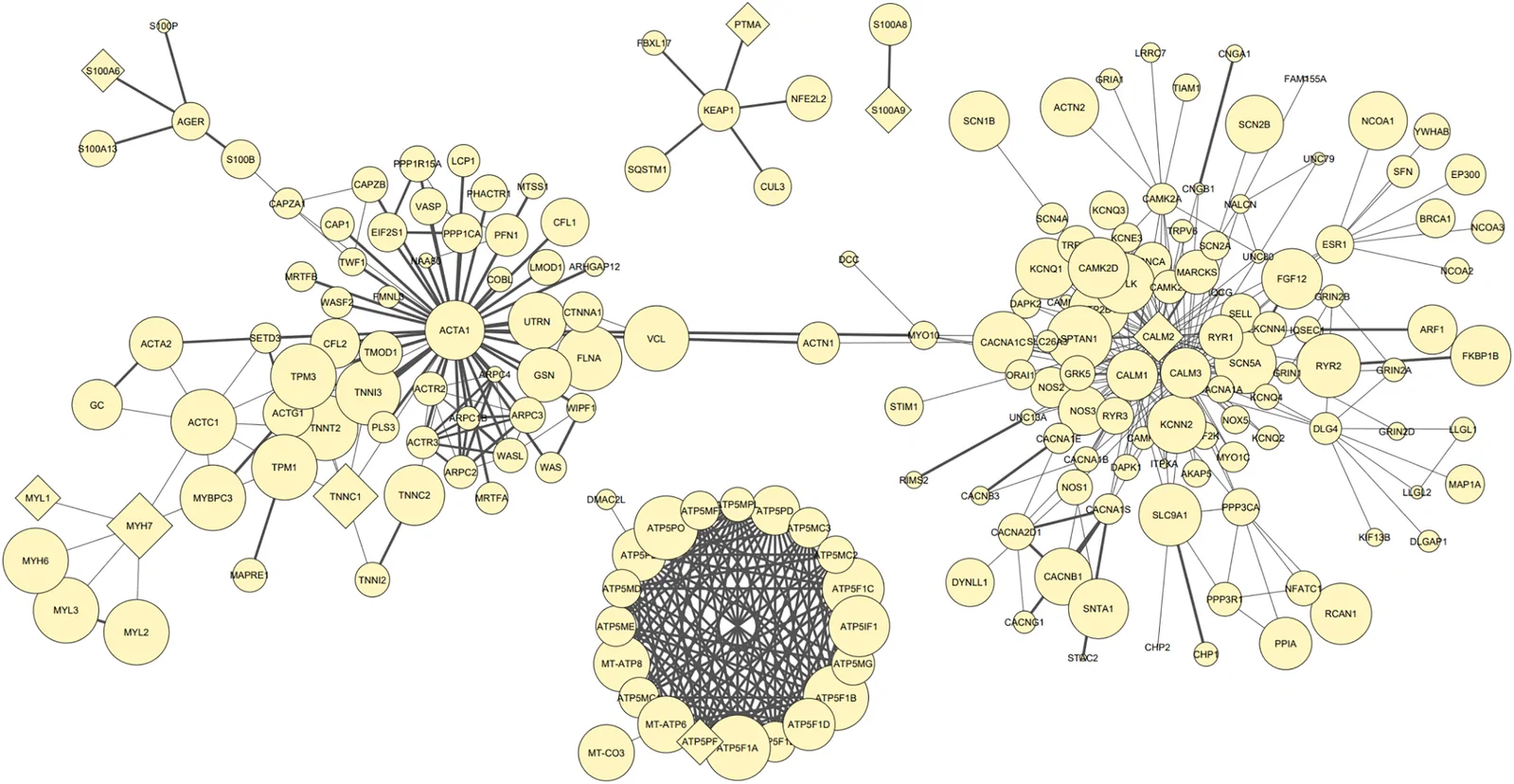
Protein-protein interaction network. The protein-protein interaction network consists of 196 proteins, represented by yellow nodes, and 477 protein interactions. The eight diamond-shaped nodes represent seed proteins, identified as the differentially expressed proteins from the original dataset. Node size corresponds to its STRING heart tissue score, with larger nodes indicating higher relative expression. The width of the interaction edges reflects edge betweenness, or the relative importance of each interaction in the network. This network demonstrates the connectivity and potential relationships among proteins perturbed by GCR_5-ion_.

**Fig 3:**
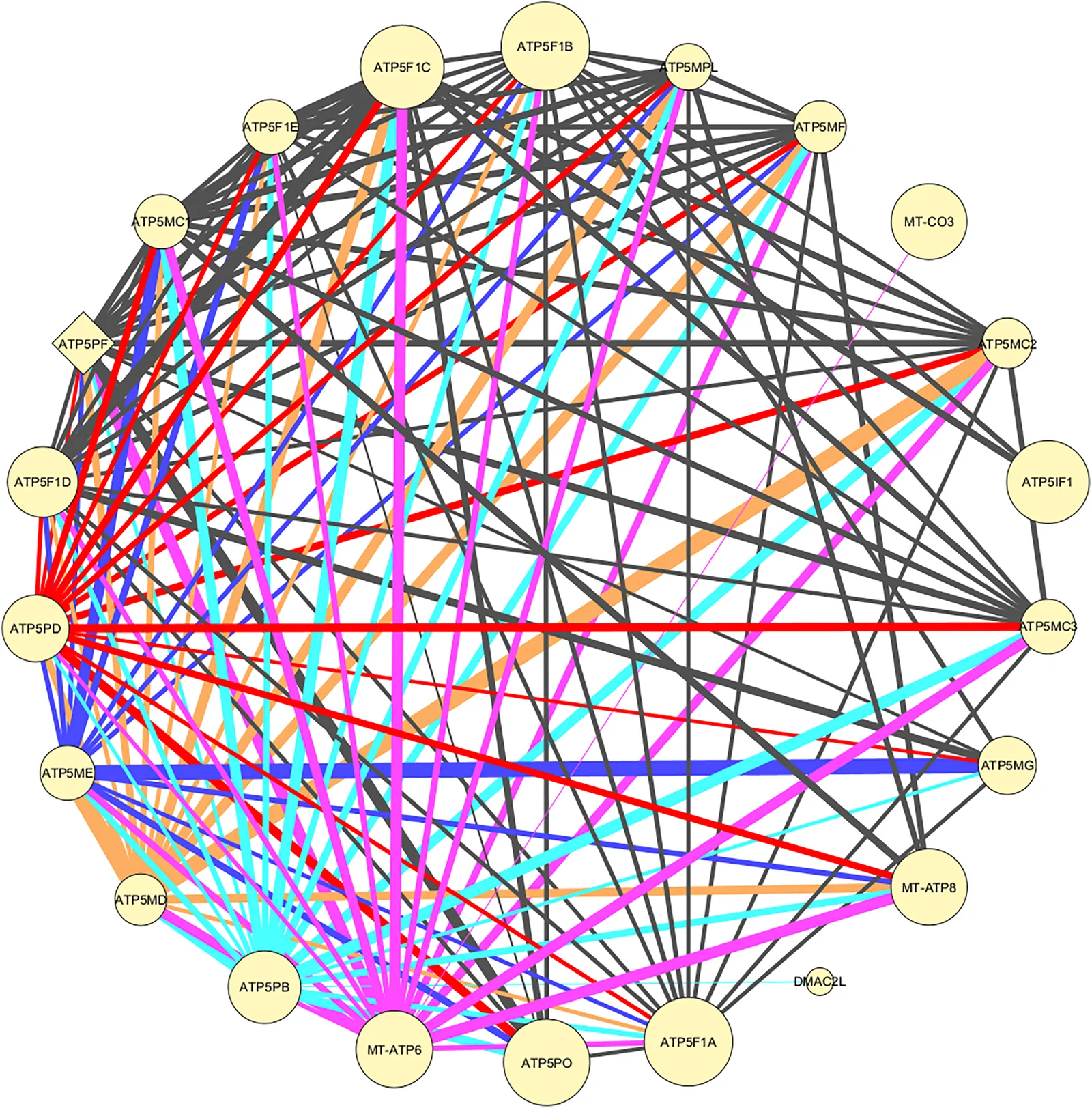
Protein-protein interaction network cluster containing ATP synthase and oxidative phosphorylation components. Cluster 2 of the protein-protein interaction network contains 22 proteins, including the seed protein ATP5PF, that are associated with ATP synthase and oxidative phosphorylation. Proteins are arranged in a circular layout based on betweenness centrality, starting with MT-ATP6 with the highest betweenness centrality and descending in a clockwise fashion. The five proteins with the highest betweenness centrality contain colored edges for differentiation. Node size corresponds to its STRING heart tissue score, with larger nodes indicating higher relative expression. The width of the interaction edges reflects edge betweenness, or the relative importance of each interaction in the network. This cluster represents the interconnected mitochondrial proteins prioritized for drug-target analysis.

Of the top 20 DEPs, 18 have human orthologs (**Supplementary Table S1 in** S1 File). The PPI, visualized in Fig 2, consists of 196 nodes and 477 edges (**Supplementary Table S2 in** S1 File). Only 8 of the 18 DEPs with human orthologs were mapped in the PPI based on the pre-specified network criteria. The diamond nodes are the original DEPs and the node sizes are arranged based on each protein’s STRING heart tissue score, meaning larger nodes are expressed more in the heart. The width of the edges are continuously mapped by edge betweenness, meaning thicker edges have a higher edge betweenness. After applying the MCL algorithm cluster of the PPI, there were 17 clusters. Our cluster of interest was cluster number two, comprised of 22 ATP synthase and oxidative phosphorylation proteins, visualized in Fig 3 **(Supplementary Table S3 in** S1 File). The proteins in this cluster are arranged in a circular layout, clockwise, by the highest betweenness centrality. The top five of these proteins in descending order, depicted by the five colored edges, are: MT-ATP6, ATP5PB, ATP5MD, ATP5ME, and ATP5PD. The top six proteins within the cluster with the highest node degree in descending order are: ATP5PB, ATP5PD, ATP5F1B, MT-ATP6, ATP5F1D, and ATP5PF. In addition, cluster number five was scrutinized because of its potential involvement in oxidative stress response **(Supplementary Table S3 in** S1 File).

### Functional enrichment analysis of the protein-protein interaction network

A functional enrichment analysis of a PPI can provide a mechanistic understanding about the genes associated with our proteins of interest. Of the GO analysis, there were 142 terms for biological processes, 81 terms for cellular components, and 66 terms for molecular functions. The top 10 GO terms, based on FDR, from each category are visualized in S2 Fig (Supplementary Table S4 in S1 File). Notable biological processes include ATP synthesis coupled proton transport, cardiac muscle contraction, and hydrogen ion transmembrane transport. Notable cellular components include mitochondrial proton-transporting ATP synthase complex, actin cytoskeleton, and sarcomere. Notable molecular functions include calmodulin binding, actin binding, actin filament binding, and proton-transporting ATP synthesis activity, rotational mechanisms.

Of the KEGG pathway analysis, there were 56 pathways. The top 10 pathways, based on FDR, are visualized in S3 Fig (Supplementary Table S4 in S1 File). Notable pathways include adrenergic signaling in cardiomyocytes, hypertrophic cardiomyopathy, dilated cardiomyopathy, cardiac muscle contraction, oxytocin signaling pathway, and calcium signaling pathway.

Of the DisGeNET over representation analysis, there were 69 diseases. The top 10 diseases, based on FDR, are visualized in S4 Fig (Supplementary Table S4 in S1 File). Notable diseases include hypertrophic cardiomyopathy, obstructive asymmetric septal hypertrophy, idiopathic hypertrophic subaortic stenosis, and several forms of congenital or familial myopathies.

### Drug-Protein Interaction Network

**Fig 4:**
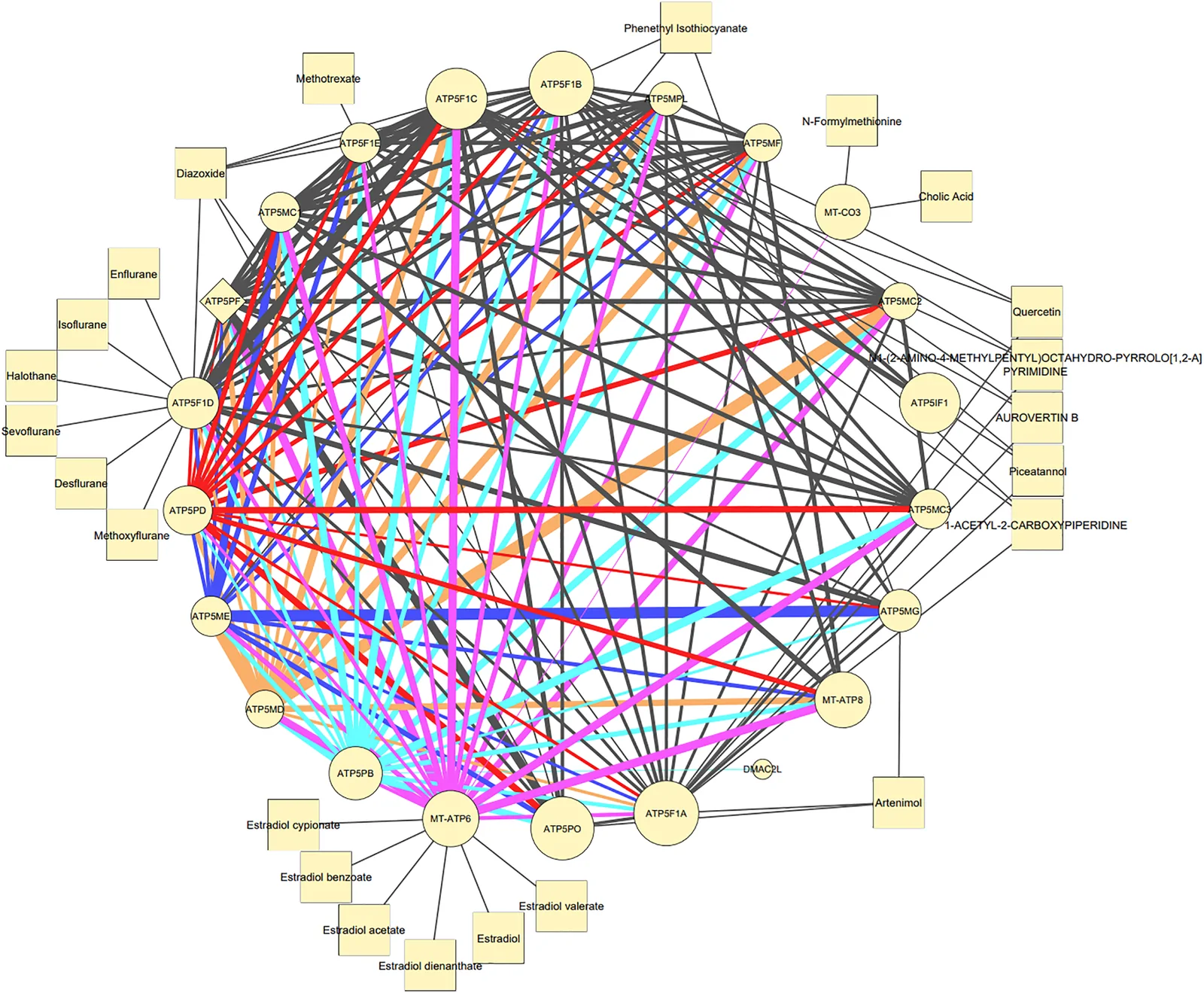
Unique drug candidates targeting the ATP synthase-enriched protein-protein interaction network cluster. Of the 23 unique drug candidates identified, seven were predicted to interact with one of the five proteins with the highest betweenness centrality, representing key topological nodes within the network. Eight drugs were predicted to interact with more than one protein within the cluster, indicating potential multi-target candidates.

The merged drug-target network contained 1357 nodes and 3141 edges. This network contained 1161 unique drug candidates, comprised of FDA approved, nutraceutical, investigational, and experimental molecules. Within the PPI cluster of interest, 23 unique drug candidates were identified based on predicted interactions with cluster proteins, as visualized in Fig 4. The drug candidates with more than one predicted target within the PPI cluster, in descending order of the number of interactions, were Diazoxide, Artenimol, Quercetin, Piceatannol, Aurovertin B, Phenethyl Isothiocyanate, 1-Acetyl-2-Carboxypiperidine, and N1-(2-amino-4-methylpentyl)Octahydro-pyrrolo[1,2-A] Pyrimidine. Of the five proteins with the highest betweenness centrality within the cluster, there were seven total drugs, six of which were various forms of estradiol and one of which was phenethyl isothiocyanate. Details about these drug candidates can be found in S2 File. All 23 drug candidates passed the Lipinski Rule-of-Five and can be found in Table 1 and Supplementary Table S5 in S1 File.

**Table 1:** Candidate Drugs from Primary Cluster of Interest.

Drug	FDA Approval	MW	HBA	HBD	LogP	Lipinski
Artenimol	N	284.16	5	1	2.21	Y
Diazoxide	Y	229.99	3	1	0.87	Y
Estradiol	Y	272.18	2	2	3.61	Y
Estradiol acetate	Y	314.19	3	1	3.6	Y
Estradiol benzoate	Y	376.2	3	1	5.04	Y
Estradiol cypionate	Y	396.27	3	1	6	Y
Estradiol dienanthate	N	496.36	4	0	8.87	Y
Estradiol valerate	Y	356.24	3	1	5.3	Y
Quercetin	N	302.04	7	5	1.19	Y
Piceatannol	N	244.07	4	4	2.49	Y
AUROVERTIN B	N	460.21	8	1	3.46	Y
N1-(2-AMINO-4-METHYLPENTYL)OCTAHYDRO-PYRROLO[1,2-A] PYRIMIDINE	N	225.22	3	2	1.34	Y
1-ACETYL-2-CARBOXYPIPERIDINE	N	171.09	3	1	−0.43	Y
N-Formylmethionine	N	177.05	4	2	−0.81	Y
Cholic Acid	Y	408.29	5	4	2.16	Y
Phenethyl Isothiocyanate	N	163.05	2	0	3.21	Y
Methotrexate	Y	454.17	9	7	−1.52	Y
Enflurane	Y	183.97	1	0	2.12	Y
Isoflurane	Y	183.97	1	0	2.22	Y
Halothane	Y	195.89	0	0	2.35	Y
Sevoflurane	Y	200.01	1	0	2.14	Y
Desflurane	Y	168	1	0	1.67	Y
Methoxyflurane	Y	163.96	1	0	2.23	Y

*MW = Molecular Weight; HBA = Hydrogen Bond Acceptor; HBD = Hydrogen Bond Donor*

### Molecular docking

Of the 15 macromolecules identified during initial RCSB PDB screening, three-dimensional structures were only available for eight of them. Based on the prespecified docking criteria, six macromolecular structures were used for molecular docking: Human ATP synthase state 1 subregion 3 (8H9F), Human ATP synthase state 2 subregion 3 (8H9J), Human ATP synthase state 3a subregion 3 (8H9M), Human ATP synthase state 3b subregion 3 (8H9Q), Human ATP synthase state 3 (combined) (8H9V), and Structure of human cytochrome c oxidase (5Z62). Diazoxide, phenethyl isothiocyanate, and each estradiol variation were docked with 8H9F, 8H9J, 8H9M, and 8H9Q. Diazoxide, Artenimol, Quercetin, Piceatannol, Aurovertin B, Phenethyl Isothiocyanate, 1-Acetyl-2-Carboxypiperidine, and N1-(2-amino-4-methylpentyl)Octahydro-pyrrolo[1,2-A] Pyrimidine were docked with 8H9V. N-formylmethionine and Cholic Acid were docked with 5Z62. The ligand-docking scores are in Supplementary Table S6 in S1 File. These docking results represent structural predictions intended to assist in prioritizing candidate complexes for further investigation rather than validated binding interactions.

### Molecular dynamics simulations

Of the five drug-protein complexes simulated, the most favorable estimated binding free energy was observed for Aurovertin B with 8H9V, with an estimated ∆G binding of −32.43 kcal/mol. This drug also demonstrated a more favorable estimated binding free energy with 8H9V compared to Diazoxide and Artenimol, each with an estimated ∆G binding of −21.60 and −20.77 kcal/mol, respectively. Phenethyl Isothiocyanate with 8H9Q demonstrated an estimated ∆G binding of −20.90 kcal/mol, a similar binding result observed for Diazoxide and Artenimol with 8H9V. The least favorable binding free energy was observed for Diazoxide and 8H9J, demonstrating an estimated ∆G binding of 11.17 kcal/mol. This weaker binding compared to Diazoxide’s own binding to 8H9V may reflect differences in the ATP synthase conformational state or ligand physicochemical properties, though the residue interactions were not specifically evaluated in the present analysis. The binding energies of the five drug-protein complexes are in Supplementary Table S6 in S1 File.

## Discussion

Radiation exposure is a well-known terrestrial risk factor for cardiovascular disease, primarily through accelerated atherosclerosis and chronic inflammation caused by the formation of reactive oxygen species (ROS) and reduced capacity to eliminate free radicals [28]. Although the components of GCR_5-ion_ differ from those of radiation therapy, many of the biochemical responses to this stressor may be similar. Given the unethicality of radiation studies in humans, analog experiments using animal models serve as a valuable approach to bridging the gap in knowledge regarding the multi-omic effects of space radiation [29]. Several successful cases of drug repositioning have been documented using various approaches, including minoxidil for hair loss, sildenafil for erectile dysfunction, atomoxetine for attention-deficit/hyperactivity disorder, and fingolimod for multiple sclerosis, among others [13]. Additionally, some drugs have been successfully repositioned for use in the context of radiation exposure, such as romiplostim for acute radiation sickness [29]. Khan et al. investigated the potential for repurposing statins, aspirin, metformin, and other radiosensitizing drugs for patients undergoing radiotherapy for various cancers [30]. Pertaining to space radiation, Nemec-Bakk, et al. reported that γ-Tocotrienol mitigated several effects of whole-body ^16^O radiation in mice, including impacts on cardiac function [6]. More recently, Siteni, et al. demonstrated radioprotection with metformin in murine models following exposure to GCRs [7]. Other studies have proposed angiotensin-converting enzyme inhibitors, xanthine derivatives, calcium-channel blockers, antioxidants, and other pharmacological agents as potential countermeasures for space radiation, though limitations exist for each approach [8,9,11] Several of these agents are FDA-approved for non-radiation indications and have been investigated for potential radioprotective effects through mechanisms including antioxidant and anti-inflammatory activity, modulation of cytokine signaling, attenuation of DNA damage, and mitochondrial stabilization. The 23 candidates identified in the present study similarly represent a heterogeneous group of compounds, including FDA-approved drugs, dietary supplements, nutraceuticals, and experimental molecules. Among these, diazoxide and quercetin have been associated with modulation of mitochondrial function, while phenethyl isothiocyanate and piceatannol exhibit potential antioxidant or ROS-modulating properties (**Supplementary Document S1 in S2 File**). Thus, although several candidates demonstrate mechanistic overlap with established or exploratory radioprotectors, particularly across antioxidant, mitochondrial, and stress-response pathways, they differ substantially in clinical status, route of administration, and safety profile. These differences highlight both the opportunities and practical limitations associated with repositioning such agents for spaceflight applications. The paucity of pharmacological data addressing space radiation injury, particularly cardioprotection, underscores the potential value of drug repositioning in this field, especially given the significant time and costs associated with drug discovery and development [29].

Although pharmacokinetic and pharmacodynamic parameters may be altered in microgravity environments, meaningful considerations remain for screening drug candidates for use in space. Factors such as a drug’s available dosage form, route of administration, side effect profile, storage conditions, and stability of the active pharmaceutical ingredient (API) should be evaluated based on existing data on medication use in space [29]. Therefore, an oral, systemically available drug requiring standard storage conditions and associated with minimal side effects may be preferred over other candidates [29]. The Lipinski Rule-of-Five serves as a widely used criterion for assessing the oral bioavailability of proposed drug candidates in the drug discovery process. Lipinski’s rule suggests that molecules with a molecular weight < 500, calculated log(P) < 5, hydrogen-bond donors < 5, and hydrogen-bond acceptors < 10 are likely to exhibit favorable permeation and pharmacokinetic properties [31]. Although not all marketed drugs adhere to the rule-of-five, this pharmacological filter provides a useful tool for drug screening, especially in the context of medication use in space. Notably, all 23 drug candidates identified in the cluster analysis met the Lipinski Rule-of-Five criteria, indicating physicochemical properties generally associated with oral drug likeness. However, several of the identified drug candidates are FDA-approved but are not available in oral formulations and are indicated for administration via alternative routes. For those candidates, the Lipinski Rule-of-Five would not be applicable as a screening criterion. Nevertheless, the Lipinski parameters were calculated for each drug candidate for comprehensiveness.

Pathway mapping using a network-based approach to identify potential targets of action is one of many computational strategies utilized in drug repositioning, alongside genetic associations, molecular docking, signature matching, and other methods [13]. To date, no studies have utilized perturbed proteomic data from organisms exposed to GCR_5-ion_ to explore the feasibility of drug repositioning. From the 18 DEPs, a PPI network consisting of 196 total proteins with 477 edges was constructed based on experimentally determined interactions. STRING, a comprehensive database of known and predicted protein-protein interactions across organisms, was used to establish this network. Restricting the PPI network to experimentally supported protein-protein interactions increased the confidence of the interaction foundation used for downstream analysis.

Through the identification of enriched GO terms, KEGG pathways, and DisGeNET disease associations within the PPI network, valuable insights were obtained regarding the implications of the perturbed proteins and their interactors for drug candidate screening. Many of the enriched biological processes, cellular components, and molecular functions presented in **S2 Fig** include factors that influence muscle contraction and cardiac function. Additionally, multiple constituents of ATP synthesis were identified, suggesting alterations to oxidative phosphorylation, a critical factor in cardiac performance [32]. This finding is further supported by the enriched KEGG pathways in **S3 Fig**, which include various cardiomyopathies and pathways related to cardiomyocyte contraction. The presence of multiple cardiomyopathy-related DisGeNET disease associations in **S4 Fig** was biologically concordant with the cardiac source of the original proteomic dataset and previously reported cardiovascular findings in Kidane et al [5].

Among the 17 clusters generated by the MCL algorithm within the PPI network, the primary interest was directed toward the cluster containing several ATP synthase and oxidative phosphorylation components. In addition to the enriched GO terms, KEGG pathways, and DisGeNET diseases, this cluster was of particular interest due to recent multi-omic evidence suggesting mitochondrial stress as a key factor in spaceflight-related health risks [33]. Further supporting this interest were cardiovascular phenotype findings reported by Bishawi et al [4]. Of note, nuclear factor erythroid 2-related factor 2 (NFE2L2) was identified in a separate cluster. This transcriptional factor is involved in the regulation of oxidative stress and antioxidant response, and a shortlist of drug candidates was compiled for its respective cluster as well (**Supplementary Table S1 in** **S1 File**). A total of 267 drug candidate interactions were identified for NFE2L2.

Lai et al. used cryoelectron microscopy to present the molecular structure of human ATP synthase in three rotational states and one substate [34]. These conformations enhance our understanding of the mechanism of ATP synthase action, revealing the coordination of the binding and release of ADP [34]. Based on the findings from Lai et al., the ATP synthase macromolecule is comprised of many protein subunits. Overall, the subunits present in these macromolecules represent a majority of the proteins identified in the PPI network cluster of interest, supporting the prioritization of these ATP synthase-associated proteins for exploratory drug-target analysis. These findings are hypothesis-generating and require computational characterization and experimental validation to determine any functional outcomes.

Network topological parameters and trends within the cluster were analyzed to identify influential proteins that may serve as drug targets (**Supplementary Table S1 in S1 File**). The first trend observed was the number of overlapping drugs and proteins. Diazoxide was found to have a predicted interaction with six different proteins within the cluster, followed by Artenimol, Quercetin, Piceatannol, Aurovertin B, Phenethyl Isothiocyanate, 1-Acetyl-2-Carboxypiperidine, and N1-(2-amino-4-methylpentyl)Octahydro-pyrrolo[1,2-A] Pyrimidine, each interacting with three different cluster proteins. The predicted interactions of these drug candidates with multiple ATP synthase and oxidative phosphorylation components suggest potential relationships with mitochondrial ATP pathways, but the functional significance of these predictions requires computational characterization and experimental validation. Additionally, drug predictions were assessed for the five clustered proteins with the highest betweenness centrality. Interestingly, six variations of estradiol were predicted to interact with MT-ATP6, the protein with the highest betweenness centrality. Each of these estradiol variations provides a similar pharmacodynamic effect in humans but differs structurally in the functional group on C3 or C17. Another protein within the top five betweenness centrality values, ATP5ME, was predicted to interact with phenethyl isothiocyanate.

Molecular docking and molecular dynamics simulations provided exploratory quantitative estimates of predicted drug-macromolecular interactions between select drug candidates and human ATP synthase conformations. Aurovertin B with combined Human ATP synthase state 3 (8H9V) demonstrated the most favorable estimated binding free energy (ΔG = −32.43 kcal/mol). Diazoxide, one of the few FDA-approved candidates identified and the drug with the greatest number of predicted protein interactions within the PPI cluster, including interactions with proteins of high betweenness centrality and node degree, demonstrated variable estimated binding free energies across ATP synthase conformational states. These differences may reflect variation in drug properties and macromolecular conformations, but the present analyses did not explore binding specificity, residue interactions, comprehensive thermodynamics, or, importantly, biological activity.

### Drug repositioning to identify space radiation countermeasures

The use of proteomic data to establish a PPI network, functional enrichment analysis, clustering algorithm, topological network parameters, drug-target network, and molecular docking in this study provides the foundation of a computational strategy for repositioning drugs for unique patient populations. The drug candidates identified are not definitive space radiation countermeasures but rather predictions based on prior research. The chemical, pharmacological, and clinical utility of these agents should be carefully evaluated within the context of the target patient population and environment. Future studies should aim to replicate these findings to build a more robust pool of drug candidates or investigate the use of these pharmaceuticals in cell lines or animal models to validate or refute the predictions.

Ultimately, the computational drug repositioning strategy presented may serve as a valuable approach for future multi-omics spaceflight studies, accelerating the prioritization of candidate pharmacological countermeasures to maintain astronaut health and performance.

### Limitations

As described by Bishawi et al., the perturbed DEPs analyzed in this study represent changes in murine heart tissue observed eight months after exposure to 150 cGy of GCR_5-ion_. This radiation dose is precisely double the maximum predicted exposure for astronauts on a mission to and from Mars (50–75 cGy) [5]. Additionally, these DEPs were collected from murine models; although the proteins were mapped to human orthologs, the findings may not fully translate to humans. While proteomic data have been collected from astronauts, numerous confounding factors in the microgravity environment complicate definitive conclusions regarding the specific effects of space radiation [1].

Only the top 20 DEPs from heart tissue were included in this analysis, representing a miniscule subset of the total DEPs reported by Bishawi et al. and Kidane et al. As a result, the PPI network constructed in this study may provide a limited or biased depiction of the broader dataset. Furthermore, the cellular, molecular, and biological effects of space radiation remain incompletely understood, posing a long-term challenge in identifying all proteins and genes influenced by this stressor within the context of computational drug repositioning.

A limitation of the molecular docking approach was the absence of several experimentally determined three-dimensional macromolecular structures, likely due to insufficient structural characterization in the literature. In this study, only select macromolecular conformations of human ATP synthase were available in the Protein Data Bank, restricting docking analyses to these specific structures. Computationally modeled structures, such as those generated through homology modeling, were not incorporated, which ultimately limited the conformational space evaluated. In addition, the docking results were not benchmarked using co-crystallized ligands or other experimentally characterized reference ligands serving as positive controls. Lastly, the binding poses were not evaluated for key residue interactions or functional binding-site occupancy. Therefore, the docking scores should be interpreted as hypothesis-generating estimates rather than evidence of validated drug-protein interactions. The molecular dynamics simulations were similarly intended as exploratory assessments of selected complexes. These simulations were performed for 5–10 ns, which is insufficient to draw conclusions regarding long-term conformational stability and thermodynamic behavior. The estimated binding free energies should therefore be interpreted cautiously. Future studies may expand on this framework through structural modeling of molecular targets, validated docking methodologies, residue interaction analyses, pharmacokinetic and ADMET characterization, longer and more comprehensive molecular dynamics simulations, and subsequent experimental validation.

## Supporting information

S1 Fig Distribution of Log2 fold-changes among significant proteins. Histogram of Log2 fold-changes for proteins meeting the significance criteria in Kidane et al. (adjusted p-value ≤ 0.05 and absolute Log2 fold-change ≥ 0.263). Most significant proteins (~72%) showed modest changes below 1.3-fold, while the top 20 proteins (~28%), selected for the largest absolute fold-changes, are highlighted (blue = upregulated; forest green = downregulated). The rug along the x-axis shows −log10(adjusted p-values), with a yellow-to-red gradient indicating increasing significance.
(TIF)

S2 Fig Gene Ontology enrichment analysis of the protein-protein interaction network. Top Gene Ontology terms for biological processes, cellular components, and molecular functions based on false discovery rate. The bar length represents the number of proteins associated with each term, and the color represents –log10(FDR).
(TIF)

S3 Fig KEGG pathway enrichment analysis of the protein-protein interaction network. The top enriched KEGG pathways based on false discovery rate. Fold enrichment is displayed on the x-axis, point size represents the number of associated proteins, and color represents –log10(FDR).
(TIF)

S4 Fig DisGeNET disease enrichment analysis of the protein-protein interaction network. Top enriched DisGeNET disease associations based on false discovery rate. Fold enrichment is displayed on the x-axis, point size represents the number of associated proteins, and color represents –log10(FDR).
(TIF)

S1 File Supplementary data supporting the protein-protein interaction network, functional enrichment, drug-target, and structural analyses. An excel workbook containing seven worksheets. S1 contains the differentially expressed proteins; S2 contains the protein-protein interaction network nodes, edges, and experimentally determined interactions; S3 contains the MCL cluster proteins, network topology, and predicted drug interactions; S4 contains the functional enrichment results; S5 contains the candidate drug characteristics and predicted protein interactions within MCL cluster 2; S6 contains the molecular docking and molecular dynamics simulations results from MCL cluster 2; S7 contains the drug-target interactions associated with MCL cluster 5.
(XLSX)

S2 File Supplementary computational methods and drug candidate information. Additional information describing the software, web-tools, and parameters used during analysis. Also, a literature-based review of selected candidate drugs identified through the drug repositioning analysis.
(DOCX)
