# Supplementary material for: Leveraging bioinformatics approaches for drug repositioning in space radiation protection
Source: PLoS One. 2026 Sep 15;21(9):e0357889. doi: 10.1371/journal.pone.0357889 (PMC13577336; doi:10.1371/journal.pone.0357889)
Supplement: Supplementary file 1 [file pone.0357889.s006.docx]

***Software and Web-tool Versions***

| **Tool** | **Parameters** |
| --- | --- |
| STRING version 12.0 | Proteins queried using “multiple proteins” option, with *Homo sapiens* selected as the organism. In the STRING settings, “active interactions sources” were restricted to “experiments,” using the highest confidence score (0.900). The maximum number of interactors was set to 100 for both the first and second shell. “Singletons” were removed. The network was extracted as a TSV file |
| Cytoscape version 3.10.2 | TSV files of the PPI were uploaded to cytoscape. The “analyze network” tool was used for topological parameters. GraphML files of the drug-protein networks were individually uploaded to cytoscape. Using the Cytoscape merge tool, the five drug-protein interaction networks were combined via an advanced network union merge. All duplicate edges and self-loops were removed. |
| clusterMaker2 version 2.3.4 | Markov Clustering (MCL) Algorithm, with a granularity parameter of two, an array source of “experimentally determined interactions,” and an edge cutoff of 0.9 |
| Drugst.One standalone tool (https://drugst.one/standalone) | The PPI proteins were queried in Drugst.One using five embedded drug-target databases: (i) DrugBank; (ii) DrugCentral; (iii) NeDRex; (iv) DGIdb); (v) ChEMBL. Each query was done separately, and the “autofill interactions” option was disabled. The five resulting drug-protein interaction networks were extracted from Drugst.One as a GraphML file |
| CB-Dock2 (http://clab.labshare.cn/cb-dock/) | 3-D conformations of the protein structure in PDB format and the drug in SDF format were queried in CB-Dock2. The number of cavities was kept at the default of 5, and the drug-protein complex was docked using the auto blind docking tool. |

***Drug Candidates***

**Diazoxide**

Diazoxide is an oral non-diuretic benzothiadiazine derivative that is commonly used for the management of hypoglycemia.^1^ It is approved by the FDA for hyperinsulinism, and has also been used off-label intravenously for hypertensive emergencies. It is an inhibitor of the sulfonylurea receptor 1 subunit of ATP-sensitive potassium channels on pancreatic beta cells, resulting in an increased permeability of potassium ions and thus hyperpolarization .^1^ Diazoxide is only commercially available in the United States as an oral suspension, making it a less than ideal candidate for use in a microgravity environment. However, an oral capsule formulation is available in Canada. Although its pKa is 8.5, diazoxide has a high oral bioavailability with an onset of action of one hour.^1^ Diazoxide is highly protein-bound, has an elimination half-life of 24 to 36 hours when taken orally in adults, and is primarily renally excreted.^1^ It must be stored at 2-30 Celsius and should remain protected from light and extreme temperature excursions.^1^

Diazoxide may have cardioprotective effects due to its affinity for mitochondrial ATP-sensitive potassium channels. In-vitro studies in animal cardiomyocytes demonstrate its selectivity for mitochondrial ATP-sensitive potassium channels rather than sarcolemmal ATP-sensitive potassium channels.^2,3^ By increasing potassium influx in myocardial mitochondria via the ATP-sensitive potassium channels, a downstream matrix swelling, mild uncoupling, and matrix alkalinization may ultimately activate cardioprotective signaling pathways.^4^ Several studies provide evidence of diazoxide’s role in protecting the function and integrity of myocardial mitochondria during and after hypoxia and oxidative stress by reducing ROS generation.^5–9^ In the context of radiation, one study found diazoxide attenuated ultraviolet radiation induced keratinocyte cell death.^10^

**Artenimol**

Artenimol, or dihydroartemisinin, is an artemisinin derivative that is often used in combination with piperaquine as an antimalarial agent.^11^ Its inactive form, artesunate, is the only FDA approved derivative and is available as an intravenous injection for the treatment of severe malaria.^12^ While its mechanism of action is not entirely understood, it is hypothesized to inhibit protein synthesis and cause oxidative stress within plasmodium species, causing cellular death.^12^ Artesunate is highly protein-bound and is activated into artenimol; the active artenimol component has an elimination half-life of 1.3 hours.^12^ It must be stored at room temperature and should remain protected from light and extreme temperature excursion.^12^

There does not seem to be a role for artemisinin derivatives in cardio or radioprotection, and evidence suggests it may actually increase ROS and enhance radiosensitivity in cancer cells.^13–19^ However, one study found artesunate treatment may ameliorate ultraviolet irradiation induced skin photoaging.^20^

**Quercetin**

Quercetin is a flavonoid commonly found in plants, foods, and supplements and may exhibit an antioxidant and anti-inflammatory effect.^21,22^ Since it is not FDA approved, the extent of its safety, efficacy, pharmacokinetics, and optimal storage conditions have not been well established. It is available as an oral, over-the-counter capsule or caplet.^22^

Its antioxidant activity are hypothesized to be due to its catechol-type B-ring and hydroxyl substitution location, which are optimal for free radical scavenging.^22,23^ For this reason, some evidence suggests that quercetin, and quercetin containing compounds, may alter radiosensitivity and offer radioprotective effects against various forms of radiation.^24–26^ When used as pretreatment in lymphocytes exposed to varying doses of gamma-radiation (1-4 Gy), it was found to significantly decrease genetic damage and thiobarbituric acid reactive substances.^24^ Similarly, various quercetin-containing compounds extracted from the pepper fruit *Capsicum annuum L* demonstrated radioprotective effects in human cell lymphocytes after exposure to X-radiation (2 Gy).^26^ In cancer cell lines and cancer xenografted mice, quercetin use induced radio-sensitization in tumors.^25^

**Piceatannol**

Piceatannol is a naturally occurring, stilbene, resveratrol analog that is found in various plants and fruits, but is considered an experimental molecule and is unavailable for clinical use in the United States.^27^ There is an abundance of evidence for its use as an anti-inflammatory molecule and an effective antioxidant with free radical scavenging activity, likely due to its many hydroxyl and methine groups.^28,29^ This may suggest its potential to preserve or restore cardiac function after exposure to oxidative stress, as demonstrated in in-vitro and in-vivo studies.^29^ Piceatannol does not exhibit favorable radioprotective effects against ionizing radiation,^30^ but one study demonstrated its ability to reduce ultraviolet-induced ROS generation in keratinocytes.^31^

**Aurovertin B**

Aurovertin B is a polyene polyketide experimental molecule that was originally isolated from the fungus *calcarisporium arbuscula*.^32,33^ It is currently unavailable for clinical use in the United States, but has a spectrum of unique biochemical activity through its mechanism of action as an uncompetitive and nonselective inhibitor of ATP synthase.^33^ In the setting of ischemia and oxidative stress, ATP synthase hydrolyzes ATP rather than synthesizing it, causing a detrimental downstream effect.^34^ Given Aurovertin B’s nonselective nature, studies demonstrate that it inhibits ATP synthase in both ischemic and healthy myocardial mitochondria.^35,36^ Therefore, it will likely not be beneficial in those with damaged myocardial tissue because it may also prevent ATP production in healthy cardiac tissue.

**Phenethyl Isothiocyanate**

Phenethyl Isothiocyanate is a natural compound commonly found in cruciferous vegetables. It is an investigational molecule that has been studied in the prevention of cancer due to its potential role in inhibiting carcinogenesis and tumorigenesis.^37^ Isothiocyanates have demonstrated antioxidant properties, likely due to their highly reactive, electrophilic, center carbon atom that is susceptible to nucleophilic attack.^38^ Phenethyl isothiocyanate has demonstrated an enhanced response to ionizing radiation in breast cancer cells,^39^ as well as protection against ultraviolet radiation-induced oxidative stress in human skin cells.^40^

**1-Acetyl-2-Carboxypiperidine**

1-Acetyl-2-Carboxypiperidine is an experimental molecule. There is currently no data on its use.

**N1-(2-amino-4-methylpentyl)Octahydro-pyrrolo[1,2-A] Pyrimidine**

N1-(2-amino-4-methylpentyl)Octahydro-pyrrolo[1,2-A] Pyrimidine is an experimental molecule. There is currently no data on its use.

**Estradiols**

Estradiol is a major, pleiotropic, endogenous hormone which regulates the transcription of many genes through binding to nuclear receptors in estrogen-responsive tissue.^41^ Through its endogenous mechanism of action, estradiol alters mitochondrial morphology and function.^42^ Evidence suggests that estradiol exposure may induce ROS mediated signaling and ultimately contribute to the progression of cancers.^43^ One study suggests a radioprotective effect of estradiol injections in mice prior to cobalt-60 radiation,^44^ but overall there is no compelling evidence to suggest its benefit. Although estrogen therapy may confer cardiovascular benefit, they carry a risk of thrombosis.^45^ In light of the case report of a female astronaut developing a venous thrombosis during spaceflight, in addition to surmounting evidence of hypercoagulopathy, venous stasis, and endothelial dysfunction in microgravity, estrogen therapy may not be an ideal option in this environment.^46,47^ However, based on a study that analyzed the list of medications routinely stored in the international space station, a combination estradiol product seems to be available for use.^48^ Its indication in this context is unclear, but it is likely for medically induced amenorrhea in female astronauts.^49^

**REFERENCES**

1.	AHFS Drug Information. Diazoxide. Published online April 15, 2019.

2.	Cardioprotective Effect of Diazoxide and Its Interaction With Mitochondrial ATP-Sensitive K+ Channels | Circulation Research. Accessed September 21, 2024. https://www.ahajournals.org/doi/10.1161/01.res.81.6.1072?url_ver=Z39.88-2003&rfr_id=ori:rid:crossref.org&rfr_dat=cr_pub%20%200pubmed

3.	Liu Y, Sato T, O’Rourke B, Marban E. Mitochondrial ATP-Dependent Potassium Channels. *Circulation*. 1998;97(24):2463-2469. doi:10.1161/01.CIR.97.24.2463

4.	Costa ADT, Quinlan CL, Andrukhiv A, West IC, Jabůrek M, Garlid KD. The direct physiological effects of mitoKATP opening on heart mitochondria. *Am J Physiol-Heart Circ Physiol*. 2006;290(1):H406-H415. doi:10.1152/ajpheart.00794.2005

5.	Deja MA, Golba KS, Malinowski M, et al. Diazoxide Provides Maximal KATP Channels Independent Protection if Present Throughout Hypoxia. *Ann Thorac Surg*. 2006;81(4):1408-1416. doi:10.1016/j.athoracsur.2005.11.045

6.	Ozcan C, Bienengraeber M, Dzeja PP, Terzic A. Potassium channel openers protect cardiac mitochondria by attenuating oxidant stress at reoxygenation. *Am J Physiol-Heart Circ Physiol*. 2002;282(2):H531-H539. doi:10.1152/ajpheart.00552.2001

7.	Dzeja PP, Bast P, Ozcan C, et al. Targeting nucleotide-requiring enzymes: implications for diazoxide-induced cardioprotection. *Am J Physiol-Heart Circ Physiol*. 2003;284(4):H1048-H1056. doi:10.1152/ajpheart.00847.2002

8.	Pharmacological preconditioning by diazoxide downregulates cardiac L‐type Ca2+ channels - González - 2010 - British Journal of Pharmacology - Wiley Online Library. Accessed September 21, 2024. https://bpspubs.onlinelibrary.wiley.com/doi/10.1111/j.1476-5381.2010.00960.x

9.	Wei K, Min S, Long C. Cardioprotective effects of mitochondrial KATP channels activated at different time. *Chin Med J (Engl)*. 2004;117(5):647-651.

10.	Cao C, Healey S, Amaral A, et al. ATP-sensitive potassium channel: A novel target for protection against UV-induced human skin cell damage. *J Cell Physiol*. 2007;212(1):252-263. doi:10.1002/jcp.21026

11.	Drugbank. Artenimol. Published online November 20, 2023.

12.	Amivas LLC (per FDA). Artesunate intravenous injection. Published online 2020.

13.	Kim SJ, Kim MS, Lee JW, et al. Dihydroartemisinin enhances radiosensitivity of human glioma cells in vitro. *J Cancer Res Clin Oncol*. 2006;132(2):129-135. doi:10.1007/s00432-005-0052-x

14.	Zhang L, Cheng LQ, Zhou Z, Lv LL, Liu GX. [Effects of dihydroartemisinin on radiosensitivity of Raji cells]. *Zhongguo Ying Yong Sheng Li Xue Za Zhi Zhongguo Yingyong Shenglixue Zazhi Chin J Appl Physiol*. 2017;33(5):385-390. doi:10.12047/j.cjap.5565.2017.093

15.	Luo J, Chen X, Chen G, et al. Dihydroartemisinin induces radiosensitivity in cervical cancer cells by modulating cell cycle progression. *Saudi Med J*. 2013;34(3):254-260.

16.	Luo J, Zhu W, Tang Y, et al. Artemisinin derivative artesunate induces radiosensitivity in cervical cancer cells in vitro and in vivo. *Radiat Oncol Lond Engl*. 2014;9:84. doi:10.1186/1748-717X-9-84

17.	Reichert S, Reinboldt V, Hehlgans S, Efferth T, Rödel C, Rödel F. A radiosensitizing effect of artesunate in glioblastoma cells is associated with a diminished expression of the inhibitor of apoptosis protein survivin. *Radiother Oncol J Eur Soc Ther Radiol Oncol*. 2012;103(3):394-401. doi:10.1016/j.radonc.2012.03.018

18.	Zhao Y, Jiang W, Li B, et al. Artesunate enhances radiosensitivity of human non-small cell lung cancer A549 cells via increasing NO production to induce cell cycle arrest at G2/M phase. *Int Immunopharmacol*. 2011;11(12):2039-2046. doi:10.1016/j.intimp.2011.08.017

19.	Fei Z, Gu W, Xie R, Su H, Jiang Y. Artesunate enhances radiosensitivity of esophageal cancer cells by inhibiting the repair of DNA damage. *J Pharmacol Sci*. 2018;138(2):131-137. doi:10.1016/j.jphs.2018.09.011

20.	Tian L, Ke D, Hong Y, et al. Artesunate treatment ameliorates ultraviolet irradiation-driven skin photoaging via increasing β-catenin expression. *Aging*. 2021;13(23):25325-25341. doi:10.18632/aging.203749

21.	Boots AW, Haenen GRMM, Bast A. Health effects of quercetin: From antioxidant to nutraceutical. *Eur J Pharmacol*. 2008;585(2):325-337. doi:10.1016/j.ejphar.2008.03.008

22.	NatMed. Quercetin. Published online February 7, 2024.

23.	Harwood M, Danielewska-Nikiel B, Borzelleca JF, Flamm GW, Williams GM, Lines TC. A critical review of the data related to the safety of quercetin and lack of evidence of *in vivo* toxicity, including lack of genotoxic/carcinogenic properties. *Food Chem Toxicol*. 2007;45(11):2179-2205. doi:10.1016/j.fct.2007.05.015

24.	Devipriya N, Sudheer AR, Srinivasan M, Menon VP. Quercetin ameliorates gamma radiation-induced DNA damage and biochemical changes in human peripheral blood lymphocytes. *Mutat Res Toxicol Environ Mutagen*. 2008;654(1):1-7. doi:10.1016/j.mrgentox.2008.03.003

25.	Lin C, Yu Y, Zhao H guan, Yang A, Yan H, Cui Y. Combination of quercetin with radiotherapy enhances tumor radiosensitivity in vitro and in vivo. *Radiother Oncol*. 2012;104(3):395-400. doi:10.1016/j.radonc.2011.10.023

26.	Materska M, Konopacka M, Rogoliński J, Ślosarek K. Antioxidant activity and protective effects against oxidative damage of human cells induced by X-radiation of phenolic glycosides isolated from pepper fruits *Capsicum annuum* L. *Food Chem*. 2015;168:546-553. doi:10.1016/j.foodchem.2014.07.023

27.	Piotrowska H, Kucinska M, Murias M. Biological activity of piceatannol: leaving the shadow of resveratrol. *Mutat Res*. 2012;750(1):60-82. doi:10.1016/j.mrrev.2011.11.001

28.	Zheng YZ, Chen DF, Deng G, Guo R, Fu ZM. The antioxidative activity of piceatannol and its different derivatives: Antioxidative mechanism analysis. *Phytochemistry*. 2018;156:184-192. doi:10.1016/j.phytochem.2018.10.004

29.	Al-Jaber HI, Shakya AK, Al-Qudah MA, et al. Piceatannol, a comprehensive review of health perspectives and pharmacological aspects. *Arab J Chem*. 2024;17(9):105939. doi:10.1016/j.arabjc.2024.105939

30.	Fabre KM, Saito K, DeGraff W, et al. The effects of resveratrol and selected metabolites on the radiation and antioxidant response. *Cancer Biol Ther*. 2011;12(10):915-923. doi:10.4161/cbt.12.10.17714

31.	Maruki-Uchida H, Kurita I, Sugiyama K, Sai M, Maeda K, Ito T. The protective effects of piceatannol from passion fruit (Passiflora edulis) seeds in UVB-irradiated keratinocytes. *Biol Pharm Bull*. 2013;36(5):845-849. doi:10.1248/bpb.b12-00708

32.	Osselton D, Baum H, Beechey R. Isolation, Purification and Characterization of Aurovertin B. *Biochem Soc Trans*. 1974;2:200-202. doi:10.1042/bst0020200

33.	Yuan S, Gopal JV, Ren S, Chen L, Liu L, Gao Z. Anticancer fungal natural products: Mechanisms of action and biosynthesis. *Eur J Med Chem*. 2020;202:112502. doi:10.1016/j.ejmech.2020.112502

34.	Capaldi RA, Aggeler R. Mechanism of the F(1)F(0)-type ATP synthase, a biological rotary motor. *Trends Biochem Sci*. 2002;27(3):154-160. doi:10.1016/s0968-0004(01)02051-5

35.	Grover GJ, Malm J. Pharmacological profile of the selective mitochondrial F1F0 ATP hydrolase inhibitor BMS-199264 in myocardial ischemia. *Cardiovasc Ther*. 2008;26(4):287-296. doi:10.1111/j.1755-5922.2008.00065.x

36.	Grover GJ, Atwal KS, Sleph PG, et al. Excessive ATP hydrolysis in ischemic myocardium by mitochondrial F1F0-ATPase: effect of selective pharmacological inhibition of mitochondrial ATPase hydrolase activity. *Am J Physiol Heart Circ Physiol*. 2004;287(4):H1747-1755. doi:10.1152/ajpheart.01019.2003

37.	Gupta P, Wright SE, Kim SH, Srivastava SK. Phenethyl Isothiocyanate: A comprehensive review of anti-cancer mechanisms. *Biochim Biophys Acta*. 2014;1846(2):405-424. doi:10.1016/j.bbcan.2014.08.003

38.	Hoch CC, Shoykhet M, Weiser T, et al. Isothiocyanates in medicine: A comprehensive review on phenylethyl-, allyl-, and benzyl-isothiocyanates. *Pharmacol Res*. 2024;201:107107. doi:10.1016/j.phrs.2024.107107

39.	Giallourou NS, Rowland IR, Rothwell SD, Packham G, Commane DM, Swann JR. Metabolic targets of watercress and PEITC in MCF-7 and MCF-10A cells explain differential sensitisation responses to ionising radiation. *Eur J Nutr*. 2019;58(6):2377-2391. doi:10.1007/s00394-018-1789-8

40.	Kleszczyński K, Ernst IMA, Wagner AE, et al. Sulforaphane and phenylethyl isothiocyanate protect human skin against UVR-induced oxidative stress and apoptosis: role of Nrf2-dependent gene expression and antioxidant enzymes. *Pharmacol Res*. 2013;78:28-40. doi:10.1016/j.phrs.2013.09.009

41.	Fuentes N, Silveyra P. Estrogen receptor signaling mechanisms. *Adv Protein Chem Struct Biol*. 2019;116:135-170. doi:10.1016/bs.apcsb.2019.01.001

42.	Klinge CM. Estrogenic control of mitochondrial function. *Redox Biol*. 2020;31:101435. doi:10.1016/j.redox.2020.101435

43.	Okoh V, Deoraj A, Roy D. Estrogen-induced reactive oxygen species-mediated signalings contribute to breast cancer. *Biochim Biophys Acta*. 2011;1815(1):115-133. doi:10.1016/j.bbcan.2010.10.005

44.	ROOKS WH II, DORFMAN RI. ESTROGEN RADIOPROTECTION IN MICE1. *Endocrinology*. 1961;68(5):838-843. doi:10.1210/endo-68-5-838

45.	Iorga A, Cunningham CM, Moazeni S, Ruffenach G, Umar S, Eghbali M. The protective role of estrogen and estrogen receptors in cardiovascular disease and the controversial use of estrogen therapy. *Biol Sex Differ*. 2017;8:33. doi:10.1186/s13293-017-0152-8

46.	Auñón-Chancellor SM, Pattarini JM, Moll S, Sargsyan A. Venous Thrombosis during Spaceflight. *N Engl J Med*. 2020;382(1):89-90. doi:10.1056/NEJMc1905875

47.	Elahi MM, Witt AN, Pryzdial ELG, McBeth PB. Thrombotic triad in microgravity. *Thromb Res*. 2024;233:82-87. doi:10.1016/j.thromres.2023.11.020

48.	Expiration analysis of the International Space Station formulary for exploration mission planning | npj Microgravity. Accessed September 21, 2024. https://www.nature.com/articles/s41526-024-00414-3

49.	Jain V, Wotring VE. Medically induced amenorrhea in female astronauts. *Npj Microgravity*. 2016;2(1):1-6. doi:10.1038/npjmgrav.2016.8
